# "The Hospital" Medico-Sociological Supplement. No. III

**Published:** 1911-03-11

**Authors:** 


### "THE HOSPITAL" MEDICO-SOCIOLOGICAL SUPPLEMENT.

(No. III.)

### THE REACTION OF PROPOSED SOCIAL REFORMS UPON OUR HOSPITAL SYSTEMS.

By BENJAMIN MOORE, M.A., D.Sc., M.R.C.S., L.R.C.P.

There is little doubt that the near future is to be fraught with many changes for the medical profession, and for all those who are interested in the progress and expansion of our great hospital systems. For better or for worse, conditions are about to change, and change enormously, and it is well to look as calmly and philosophically as possible into the future, to try to discern how affairs are moving, so as to be able to take advantage of new conditions and play our part in guiding matters aright.

At the present moment, as far as can be judged from somewhat intricate statistics, about one-fourth or somewhat less of our sick poor are treated in voluntary hospitals supported by charity and charitable endowments, and slightly over three-fourths in public institutions supported by rates or taxes and called Poor Law hospitals and municipal hospitals. There is absolutely nothing to distinguish the circumstances of the one set of these patients from the other, and nothing to distinguish either set from a third set of patients in no kind of hospital whatever, but lying sick in the slums, occasionally attended in a perfunctory and random way by overworked and underpaid Poor Law dispensary doctors, who work almost for nothing and find their own drugs and surgical dressings.

### Sick Relief for the Indigent.

All these sick and afflicted persons, numbering hundreds of thousands throughout the country, are virtually paupers, to use an unpopular word; that is to say, they are poor persons unable themselves to pay for medical or surgical attendance. If this is not so, then the medical profession is being defrauded by the hospital authorities which have admitted them. However, we shall suppose here that hospital abuse is non-existent, and take it that these hospital patients are all *bôna fide* poor persons unable to help themselves in their affliction, and dependent upon the support of others, whether that support is given by voluntary benevolence or charity, or whether it is exacted by rates or taxes from churl and benefactor alike, each paying the same number of pence in the pound.

Our theme is to consider in how far proposed legislation, such as Poor Law reform, State sickness insurance, and provision of State sanatoria for phthisis, may change our present systems for providing for ill-health in the case of these hundreds of thousands of poor people.

### Poor Law Reform.

There can be no question that adequate Poor Law reform will and must bring about enormous changes in the larger class of the Poor Law hospitals, and there can be but little doubt that this and State sickness insurance combined are bound to affect in some fashion or other the voluntary hospitals also.

Whatever faults some of us may have to find with their present organization and methods of conducting their business, the voluntary hospitals of our country are a great and flourishing national institution, which as a whole we cannot afford to lose in the flux of affairs in the medico-sociological world which is so rapidly coming upon us. It must be our bounden duty to "hold fast that which is good," and preserve one of the noblest assets of our national life, however we may outwardly have to modify it, or change the garb to suit a new fashion.

### What the Voluntary System Means.

No eulogistic description is requisite to prove that our voluntary hospitals stand for all that is best in our whole hospital system to-day, and that it is their example which has elevated some of the Poor Law institutions to that considerable level they have attained, and by the force of contrast keeps urging one Board of Guardians after another to make better provision for the sick poor. Sweeping statements are never universally true; there are, of course, some voluntary hospitals, more especially those known as special hospitals, which might almost be described as a disgrace in the feebleness of their organisation and management, and in regard to that large percentage of charitably subscribed money which becomes uselessly frittered away before it reaches the patient or ameliorates his ills. There are other large voluntary hospitals which, while showing a high level of efficiency in their work, are most inordinately expensive and extravagant as institutions intended for relieving the poor at the expense of the charitable. Contrariwise, there are many Poor Law hospitals which might now well be taken as examples in the excellent middle course they pursue in relieving the sufferings of the poor and protecting the health of the community without squandering public money upon unnecessary luxury.

### The Faults of the System.

However, taking the history of our hospital system as a whole, the guide and beacon of progress are to be found in our voluntary hospitals, and the devoted bands of laymen interested in charity and well-doing who have directed their destinies. Like all mundane institutions these voluntary hospitals are imperfect, and anyone so disposed could cast a glaring light upon places where science has progressed and left the majority of them behind, upon waste of public time and ill-arrangement of service in their out-patient departments, upon the modes of appointment of the medical staff, upon the cozening of the outside medical profession by the institution of paying wards to back up the resources of charity, or otherwise upon the free treatment and entertainment of those well able to pay, and upon a sufficiency of other abuses and anachronisms to make quite an article by themselves. All these faults are not diminishing, but increasing as time goes on, and the three great elements in the treatment of the diseases, not merely of the poor, but of us all, namely, medical science, the hospitals, and the medical profession, are growing ever more widely apart and estranged from one another. In spite of all this, however, the voluntary hospitals are the best part of the whole chaotic mass of things medical, and have maintained throughout all changes certain elements which must be preserved at all hazards for the co-ordinated system which it is to be hoped may soon emerge.

It will be of interest in the first place to enquire whether the voluntary hospitals are likely to be involved by coming changes, so as to be municipalised or nationalised, and if so can their present permanent endowments from past charitable donors be preserved and utilised, and, more important still, can the present individualistic enthusiasm and zeal for highest service to their fellow-men of their voluntary committees be maintained, or rather duly increased and expanded, as the wider sphere of work develops with new conditions.

There seems no good reason why a hopeful and satisfactory affirmative should not be given to both these queries.

### Some Signs of the Times.

First, as to the voluntary hospitals coming under municipal or national control, there are signs even now of that occurring in all those new directions of medical work for the wage-earning classes which have recently been provided by legislation. It is only the temporary and passing difficulty of arranging hospital work without defrauding the medical profession outside which has prevented much more wholesale use being made in these directions of the voluntary hospitals. It is only necessary to alter certain conventions, notably that of expecting medical men to do professional work in hospitals for nothing in a spurious and unworthy form of charity, in order to get over most of the difficulties of treating, not only school children, but also all out-patients at hospitals, by the general medical practitioners of the district.

Again, the habit is growing of voluntary hospitals, not yet upon the rates, obtaining grants from municipal sources for sites, or buildings, or equipment, and the distance to rate aid for annual upkeep is not immeasurable. More and more, municipal work and municipal money are always drifting towards the hospitals, and this fortunately is happening without causing any diminution in voluntary contributions and legacies from the wealthy.

There is no greater delusion than to suppose that because any municipal or national interest receives direct municipal or national funds that this will proportionately decrease individualistic effort and diminish the support and aid from the more fortunate wealthy citizens or inhabitants. Neither the money, nor, what is more important than money—namely, the life-long interest and applied brain-power and hard, devoted labour of the fittest and best of our citizens, will be cut off from our great institutions because they begin to receive municipal and State aid.

### The University Example.

This view is based on no mere fancy; it is a proven fact in the case of other great institutions.

Take, as an example, the history of our great municipal Universities. Most of these began their careers, a generation or more ago, as University Colleges, and at first the money was raised by voluntary contributions of wealthy and high-minded citizens, who used the means acquired by intense individualistic effort to help onward the whole social community. Later, as the knowledge of the supreme utility of Universities became more widely spread, broad-minded politicians voted Government grants to these incipient Universities, and some of the believers in purely voluntary effort looked askance, and thought and said that the springs of individual interest and private money would dry up. But, behold, they but flourished all the more, and later, when both private donations and Government grants —although each largely increased in amount with the passage of the years—became insufficient for the growth and expansion of these great seats of learning, the keenest-eyed of the rulers of the destinies of the great municipalities said, "These Universities are amongst the finest assets of all our undertakings, and if they are cramped in their expansion they must also have municipal aid." "What! put the University on the rates!" again cried the croakers; "do, and you will ruin it; you will never get private money for a rate-supported institution." But the Universities did go on the rates with full approval of the great bulk of the citizens, who appreciated the great work upon the sides of humanitarianism and applied science alike that the local University was doing; and, instead of dropping off, the local voluntary contributions have gone up by leaps and bounds, till now, with voluntary contributions, and Government grants, and rate aid combined these modern Universities are as wealthy as the more venerable halls of learning which formerly catered for a small select class of wealthy, aristocratic, or narrowly professional students. A wide-spread University training is thus working upon the whole mass of the people instead of being, as formerly, as much the property and perquisite of special classes as were the entailed acres of the landed gentry.

In almost precisely a similar fashion, the work of the voluntary hospitals can be extended if we have the wisdom to guide it aright, so that without losing anything of voluntary effort, we can superadd both State and municipal effort and endowment, and so properly cater for the entire community.

### Wanted Co-ordination and Adaptation.

Co-ordination and adaptation of method are required, and the inclusion in proper fashion of the medical profession, but none of these things are impossible, and in spite of all the present jarring and striving elements and temporarily opposed factions and interests, they are coming.

The example given of the case of the Universities can be manifolded by anyone who cares to think the matter out, and is at all familiar with the workings of municipal life. The principle is seen in our national and municipal museums and picture galleries, in our public parks, in our public libraries, in our public buildings, monuments and memorials—everywhere private munificence and municipal and State contribution existing alongside and aiding and complementing one another, in no sense antagonistic to one another. Why, therefore, should it be supposed that State or municipal aid for our hospitals will destroy individual interest or individual monetary aid?

### The Voluntary Principle in State Aid.

After all, how are our affairs of State and our municipalities conducted, if it is not by voluntary effort? There is no compulsion upon anyone to be a City Councillor or a Member of Parliament. It is but a foul aspersion on our human nature, with all its defects, to say that our ideal and incentive is money, and that we only do what we are paid for, and work only for selfish ends. Our ends may be selfish in one sense, but the community honours those who work for it, and many find their highest honour and greatest pleasure in serving the community. Individualism and socialism, in their truest senses, are not opposed, as so many false prophets teach; the two at their best are inexplicably woven together into the fabric of work of any community. Nothing national is ever accomplished save by the work of individuals, and true socialism is the team work of individuals unselfishly undertaken. What is this but the highest and noblest form of individualism?

Thus much has been said on individualism and socialism, because so many people require convincing that State aid or municipal aid is opposed to individual work or interest in an institution, and such a dangerous fallacy is likely to delay progress toward a unified scheme of hospital work and medical service.

### The Legislation of the Future.

It is seen then that recent legislation is tending to bring the voluntary hospitals nearer to subsidy from the rates; let us glance now at the probable effects of contemplated legislation.

At first sight it might seem as if the scheme of State sickness insurance, which has recently been adumbrated in a semi-official fashion in the Press, contained little or no provision which involved either the medical profession or our voluntary hospitals. A closer inspection, however, and an attempt to visualise the scheme at work soon demonstrate that both the medical profession and the voluntary hospitals are either bound to be deeply involved in it, or cease to exist as they exist to-day. This scheme must inevitably, in the course of a few years, become the basis of all medical treatment and all hospital systems catering for the industrial classes, and involving quite thirty-eight millions out of the forty-four or forty-five millions of inhabitants of this country. The scheme will draw together into unity almost the entire medical profession, and consolidate and direct the whole hospital service of the country. All this is to the good; the only pity is that instead of uniting and consolidating all the friendly societies at the same time, and placing them under Governmental control, by a process of friendly absorption and re-direction, it is proposed to leave these societies free, fighting and competing with one another for members, and so wasting money on a most expensive and inco-ordinated executive and management. It may be safely prophesied that very soon the more powerful of these friendly societies will amalgamate their businesses, and we shall have a gigantic scheme, subsidised by Government, employers and workmen, in the hands of a huge monopoly, which will have to be bought out like other monopolies, at great expense to the community. Such a wastage of time and energy is of no advantage to any one, and it is a great pity that the Government cannot see its way to nationalise the whole system now, finding room on the working staff of the new unified scheme for all the present officials of the great friendly societies. The task of arrangement is not an impossible one, and a little delay, and thought, and labour now would save endless expense and trouble in the future, when the work has all to be done over again.

### State Sickness Insurance.

What are the salient features of the new scheme of State sickness insurance, and how do these affect doctors and hospitals? It is to be proposed apparently that every employed person whose income is less than £160 must insure with some company or friendly society, and that in case of sickness the benefit shall be double that to which he, or she, would be entitled under normal business conditions; because an equal amount to the employer's contribution is also to be paid to the nominated friendly societies in equal shares by Government and employer. The minimum amount of compulsory insurance is to be such that the insured shall receive ten shillings weekly for twenty-six weeks' illness, if necessary, and, thereafter, five shillings weekly, if necessary, until the age of seventy years is reached.

Although the scheme is one of *sickness* insurance, there is no mention either of calling in a doctor or of medical or surgical treatment, at home or in hospital. It would look at first sight as if the insured person merely stopped working, reported himself as sick, and forthwith started to receive ten shillings a week, whatever (short of £160 a year) his ordinary wages might be; and that he then might proceed to spend this weekly sum as he chose, and call for medical assistance or not, as he deemed necessary.

### Some Inevitable Objections.

The first criticism which arises to one's mind is the crudity of a system which provides the same dole of sick pay, whether the wage of the recipient has been ten shillings or three pounds a week. The second is whether the economy of a system is above suspicion which leaves it to the worker's choice whether he shall have a doctor or not when he is so seriously ill that he cannot work. There is a sore temptation placed upon a worker who has been earning, say, thirty shillings a week, when he is suddenly incapacitated and placed upon ten shillings a week, to spend this upon rent and necessary food for himself and family, rather than upon medical advice and treatment. Surely, if for no other reason than national economy, adequate medical treatment ought to be provided, as well as the ten shillings a week.

When we look a little deeper into this apparently simple scheme, which is going to involve fifteen million workers at the outset, and soon will extend to involve their wives and families also, do we really find that the doctor and hospital are so completely shut out from it? Who is to certify that the worker is ill and entitled to his ten shillings a week, who is to look out that he does not continue to draw his ten shillings a week and remain out of work a little longer than necessary, and who is to look after the interests of the friendly society, and make him efficient for return to the "fighting line" as soon as possible? Evidently there must be a doctor, and equally evidently that doctor is going to be an official of a friendly society, employed by the friendly society, paid by the friendly society, and dismissible by the friendly society; and this is how the bulk of the medical work of the country is to be done, by a set of officials of rival friendly societies. It is an extension of the club system, with its disadvantages made more rampant instead of being eliminated, as they would be in a national medical system.

### The German Warning.

The only alternative is that the sick worker shall first of all be seen by a friendly society's doctor, have his cause of illness diagnosed, and be certified for sick pay. Then he calls in and pays his own private doctor, if he is so disposed, out of his ten shillings a week, and also he is visited and his condition diagnosed at intervals by the friendly society's doctor, to find out when he is fit to go back to work. So that the amount and expense of medical work are practically doubled. This is too ludicrous to conceive as a gigantic system for fifteen millions of people, so that there is little question that the friendly society's doctor will do the work, and so away goes the much cherished choice of doctor, which could be retained in a national system, and away along with it goes all independence from the medical profession. Such a scheme is now working in a most unsatisfactory way in Germany; both workmen and doctors are highly dissatisfied with it, and as soon as the doctors have a powerful enough union there is going to be a strike.

It is therefore sincerely to be hoped that in this country the Government will keep control and make the scheme a truly national one.

But, whether the expenses of sickness are looked after by Government or by friendly societies, it is clear that the economists of either system will soon learn that some means must be found by which the duration of sickness is made as short as possible, so as to diminish Government expense in the one case, or increase society profits in the other.

### Exploiting the Hospitals.

Now, in practically all surgical cases, and in all serious, or long-continued, medical cases, this will mean calling in the facilities of the hospitals. There will here be made the usual attempt to exploit charity and try to get the hospitals to give accommodation, and the medical men within them their professional services, for nothing. Such an attempt will hardly succeed, however, where fifteen millions of the population are involved; fortunately, the scale is too huge for that, and would simply mean ruin to both the medical profession and the hospitals.

What will occur under this combination of circumstances? The answer is quite obvious; the Government, or the friendly societies, as the case may be, who have been receiving the insurance funds, will have to commence to pay both for hospital maintenance and the fees or salaries of the doctors who attend to the workers in the hospitals; more work than now will be done in the hospitals; but this will not be a hardship on the medical profession, because the work will be paid for.

Such a position is bound to be reached when the scheme is at work, for suppose an insured workman who has been paying into a friendly society's funds has to be taken to hospital, there to receive rest, food, and treatment, which fit him to return sooner to work, so saving the funds of the friendly society. Then the hospital authorities have a direct claim on the society for subsidy, and it is scarcely to be anticipated, in view of the present condition of the funds of most voluntary hospitals, that such a claim can or will long be left unredeemed.

Again, at the present time, as pointed out earlier, only about one-fourth of our industrial classes when in hospitals are treated at voluntary hospitals, and three-fourths at Poor Law hospitals. Now, suppose State insurance passes into law, then, when in health, the greater part of this three-fourths would consist of the contributories to the compulsory scheme of insurance against sickness. What, it may be asked, is now going to be done with this huge body of people when their illness is so serious that they have to be sent to hospital? They are under State sickness insurance, and can therefore, when they fall ill, scarcely be pauperised and sent to a workhouse hospital. What is to become of them? They will absolutely crowd out the voluntary hospitals if sent there, so that it is obvious, in addition to subsidising the voluntary hospitals by State or friendly society, that the Poor Law hospitals must be made non-pauper institutions, and placed under State, or municipality, or friendly society, whichever is running the scheme and taking the moneys of the insured. A new spirit must therefore be infused into these new hospitals, and they must be brought up to the level of the voluntary hospitals, for it will not do in the same city to have the same paying State-insured patients sent to two different types of institution.

Here State sickness insurance and Poor Law reform coincide and reinforce each other, and together force forward a unification of our hospital system.

Not a Task for the Friendly Societies.

These further considerations also demonstrate clearly that the task involved is too huge for the friendly societies; only Government can carry it out, so many and extensive are its ramifications.

All the goodness and all the executive ability of the great friendly societies should be utilised; they have nothing to gain by a rivalry, or by being overwhelmed by the gigantic compulsory scheme being thrown upon them; they have all to gain by being absorbed and made part of a national scheme, and the present interests of members can be most carefully safeguarded in a national scheme. Similarly, all the work and energy of existent voluntary benevolent agencies, and the present committees of voluntary hospitals, can be preserved and utilised. The new hospital mechanism need not be made so rigidly municipal, or national, as to exclude voluntary aid, and can be so devised as to give scope still for private munificence, and for the living of valuable public lives by our greatest citizens. Here some lessons can surely be gathered from the way in which our Universities still retain, without losing their independence and freedom, all types of assistance, public and private; from the existence of a civilian army, of volunteers or Territorials, in addition to our paid army, and from many similar combinations and joint growths of our modern civilisation.

Even when we have State funds for hospital maintenance, and for payment of medical men, we shall still be able to retain, and in increased amount, the endowment of clinical research for advance in medical knowledge by the private munificence of the opulent citizens of our great communities. We shall then be able to find fellowships to give to young men to engage in the study in laboratory and hospital of the unsolved problems of disease; we shall be able to pay from such voluntary sources for those more expensive equipments which the advance of science are ever making more urgent, both for the treatment and the study of disease.

Nor is it money alone which we shall still require; the guiding intellect and wise counsel of voluntary committees will still be necessary when we have State subsidy and a better organisation, and this better organisation and equipment will enable voluntary enterprise to advance more rapidly, whereas it is now too often harassed by want of means and want of system.

Those who are engaged in voluntary work for hospitals, and have grown to love it as a good and useful life-work, need therefore have no fear of State insurance and unification of medical service stultifying or decreasing their sphere of usefulness; on the contrary, such changes must enormously enlarge it. Whatever may come, and whatever names be applied, the work of hospitals in the future must be carried on by the only class of people in the community who are capable of doing it, and these are they, all honour to them, who have been struggling with it in the past under a heavy handicap.

